# The Australian Baby Bonus Maternity Payment and Birth Characteristics in Western Australia

**DOI:** 10.1371/journal.pone.0048885

**Published:** 2012-11-07

**Authors:** Kristjana Einarsdóttir, Amanda Langridge, Geoffrey Hammond, Anthony S. Gunnell, Fatima A. Haggar, Fiona J. Stanley

**Affiliations:** 1 Telethon Institute for Child Health Research, Centre for Child Health Research, University of Western Australia, Subiaco, Perth, Western Australia, Australia; 2 Health and Wellness Institute, Edith Cowan University, Joondalup, Perth, Western Australia, Australia; 3 Centre for Health Services Research, School of Population Health, The University of Western Australia, Crawley, Perth, Western Australia, Australia; Tehran University of Medical Sciences, Islamic Republic of Iran

## Abstract

**Background:**

The Australian baby bonus maternity payment introduced in 2004 has been reported to have successfully increased fertility rates in Australia. We aimed to investigate the influence of the baby bonus on maternal demographics and birth characteristics in Western Australia (WA).

**Methods and Findings:**

This study included 200,659 birth admissions from WA during 2001–2008, identified from administrative birth and hospital data-systems held by the WA Department of Health. We estimated average quarterly birth rates after the baby bonus introduction and compared them with expected rates had the policy not occurred. Rate and percentage differences (including 95% confidence intervals) were estimated separately by maternal demographics and birth characteristics. WA birth rates increased by 12.8% following the baby bonus implementation with the greatest increase being in mothers aged 20–24 years (26.3%, 95%CI = 22.0,30.6), mothers having their third (1.6%, 95%CI = 0.9,2.4) or fourth child (2.2%, 95%CI = 2.1,2.4), mothers living in outer regional and remote areas (32.4%, 95%CI = 30.2,34.6), mothers giving birth as public patients (1.5%, 95%CI = 1.3,1.8), and mothers giving birth in public hospitals (3.5%, 95%CI = 2.6,4.5). Interestingly, births to private patients (−4.3%, 95%CI = −4.8,−3.7) and births in private hospitals (−6.3%, 95%CI = −6.8,−5.8) decreased following the policy implementation.

**Conclusions:**

The introduction of the baby bonus maternity payment may have served as an incentive for women in their early twenties and mothers having their third or fourth child and may have contributed to the ongoing pressure and staff shortages in Australian public hospitals, particularly those in outer regional and remote areas.

## Introduction

The declining fertility rate around the world has raised concerns in many developed countries, resulting in policies and strategies put in place to boost fertility rates [1]. The Australian government announced similar intentions in May 2004 with the introduction of the baby bonus on 1^st^ July 2004, involving a lump sum payment to all families following childbirth or the adoption of a child [2]. The bonus was revised in 2009, where it was restricted to low and middle income families and changed to bi-weekly payments [2].

The baby bonus has been reported to have increased birth rates by 12% in Western Australia (WA) [3] and 7% in New South Wales (NSW) from 2004 to 2006 [4]. The mothers reported to contribute most to this increase were mothers aged 20–29 living in the highest socio-economic (SE) areas [3], and young mothers having their second or third child [4]. Furthermore, Lain et al. has reported significant increases following the baby bonus introduction for births in public hospitals, and for vaginal deliveries, with the estimated cost of births in NSW increased by AUS $60 million [5].

In this study, we explored for the first time the influence of the Australian baby bonus introduction on the change in birth rate for mothers insured by private health insurance (private patients) and the Australian Health Care Agreement (public patients). We also investigated where the baby bonus had the greatest birth rate influence with regard to other maternal demographics and birth characteristics using data with longer follow-up than previously published research [3], [4].

## Methods

### Ethics Statement

The use of de-identified, administrative health data for this study without patient consent was approved by the Human Research Ethics Committee of the WA Department of Health. This study was performed in accordance with the Declaration of Helsinki.

### Study Data

WA birth data from the WA Midwives Notification System (MNS) from July 2001 to December 2008 was linked with the WA Hospital Morbidity Data Collection (HMDC) by the Data Linkage Branch at the WA Department of Health and provided to the researchers in de-identified form. The collection of this data is governed by legislation requiring all births in WA occurring on or after 20 weeks gestation or infants born with birth weight of at least 400 g to be registered as well as all hospital admissions and separations from all hospitals in the State.

For the study population included in this study, multiple births (e.g. twins) were counted as one birth admission, with the information on length of hospital stay for the first born twin being used. Also, both live- and stillborn infants were included. Length of stay was categorized into 0–3 days and 4+ days following birth since most mothers and babies stay less than 4 days in hospital following an uncomplicated vaginal birth.

The MNS included information on the Index of Relative Socio-Economic (SE) Advantage and Disadvantage (IRSAD) and Accessibility/Remoteness Index of Australia (ARIA+). The IRSAD values are based on information on household income, educational attainment and occupation from the Australian Census conducted every five years and assigned to each collection district area in the state. The IRSAD values were divided into sextiles for all analyses with the highest three sextiles combined to indicate high area-based SES and the lowest three combined to indicate low SES. The ARIA residential remoteness index is also calculated from Census information every five years and reflects access to services in a collection district area. It was divided into major cities, inner regional Australia, outer regional Australia, remote Australia, and very remote Australia. The IRSAD and ARIA values from the 2001 and 2006 Censuses were assigned to each birth admission based on maternal area of residence at the time of birth.

The HMDC provided information on the funding source of the mother at the time of each hospital birth. Funding source was categorized to reflect two types of mothers; those treated as public patients and those treated as private patients during delivery. Private patients were defined as those funded with private health insurance (PHI) or who were self-funded, whereas public patients included those insured under the Australian Health Care Agreements and Reciprocal Health Care Agreements. Hospitals were divided into private or public with the exception of the single obstetric tertiary hospital in WA, which was defined as tertiary.

### Statistical Analysis

We used interrupted time-series analyses to estimate the average quarterly birth rates in WA before (pre-BB) and after (post-BB) the baby bonus implementation and compared the post-BB rates with rates that would have been expected had the policy not occurred (post-2004). We calculated the birth rates shown in Table 1 (maternal demographics) from the quarterly birth counts in our data (numerators) and annual population figures for 12–50 year old females in WA (denominators) based on 5-yearly census data published by the Australian Bureau of Statistics (ABS) [6]. Both numerators and denominators were divided according to age group, SES group and remoteness group. For determination of birth rates shown in Table 2 (birth characteristics), the annual birth counts in WA were used as denominators.

**Table 1:** Average quarterly birth rates by maternal demographics before and after the baby bonus introduction as well as post-2004 assuming the policy did not occur.

	Observed pre-BBquarterly rates^a^	Observed post-BBquarterly rates^a^	Expected rates post-2004^a^	Rate difference^b^(95% CI)	Percentage difference^b^(95% CI)
All	11.2	12.5	11.0	1.4.(1.2,1.6)	12.8 (11.3,14.5)
Maternal age (years)					
12–19	3.2	3.3	2.8	0.5 (0.5,0.5)	17.3 (16.0,18.6)
20–24	15.0	15.8	12.5	3.2 (2.8,3.7)	26.3 (22.0,30.6)
25–29	26.5	27.6	24.0	3.5 (3.1,4.0)	14.8 (12.6,17.1)
30–34	27.2	30.9	31.0	−0.1 (−0.5,0.2)	−0.3 (−1.5,0.9)
35+	4.5	6.0	5.3	0.7 (0.6,0.8)	13.3 (12.6,14.1)
Area-based SES					
High^c^	9.1	11.2	10.0	1.2 (1.2,1.3)	12.1 (11.5,12.8)
Low^d^	11.7	11.5	10.4	1.1 (0.9,1.3)	11.0 (8.9,13.1)
Residential remoteness					
Low^e^	10.3	11.3	10.4	0.9 (0.8,0.9)	8.4 (7.8,8.9)
High^f^	10.2	12.0	9.1	2.9 (2.8,3.1)	32.4 (30.2,34.6)

a per 1000 population.

b between post-BB rates and expected rates post-2004 (assuming the policy did not occur).

c SES: Socio-economic status. Sextiles 1–3.

d SES: Socio-economic status. Sextiles 4–6.

e Major cities and inner regional Australia.

f Outer regional Australia, remote Australia, and very remote Australia.

BB = baby bonus.

**Table 2:** Average quarterly birth rates by birth characteristics before and after the baby bonus introduction as well as post-2004 assuming the policy did not occur.

	Observed pre-BBQuarterly rates^a^	Observed post-BBQuarterly rates^a^	Expected rates post-2004^a^	Rate difference^b^(95% CI)	Percentage difference^b^(95% CI)
Parity					
1^st^ child	45.9	47.9	47.7	0.2 (0.2,0.3)	0.5 (0.3,0.7)
2^nd^ child	32.9	31.6	32.3	−0.7 (−0.7, −0.7)	−2.1 (−2.2, −2.1)
3^rd^ child	13.5	13.1	12.9	0.2 (0.1,0.3)	1.6 (0.9,2.4)
4^th^+ child	7.7	7.4	7.3	0.2 (0.2,0.2)	2.2 (2.1,2.4)
Funding source					
Public	63.3	62.8	61.8	0.9 (0.8,1.1)	1.5 (1.3,1.8)
Private	35.1	35.1	36.7	−1.6 (−1.8, −1.4)	−4.3 (−4.8, −3.7)
Hospital type					
Public	43.9	41.9	40.4	1.4 (1.0,1.9)	3.5 (2.6,4.5)
Private	37.3	37.2	39.7	−2.5 (−2.7, −2.3)	−6.3 (−6.8, −5.8)
Tertiary	17.2	18.8	18.6	0.2 (0.1,0.4)	1.2 (0.2,2.2)
Mode of delivery					
Unassisted vaginal	57.9	53.9	52.9	0.9 (0.5,1.4)	1.8 (0.9,2.6)
Vacuum/Forceps	12.1	13.0	11.2	1.9 (1.4,2.3)	16.9 (12.7,21.0)
Caesarean with labour	10.7	12.2	11.5	0.7 (0.7,0.8)	6.5 (5.9,7.0)
Caesarean without labour	19.3	20.9	25.6	−4.7 (−6.0, −3.4)	−17.9 (−22.2, −13.5)
Length of hospital stay					
0–3 days	41.0	47.0	40.3	6.7 (5.0,8.3)	16.6 (12.5,20.8)
4+ days	59.0	53.0	59.7	−6.7 (−8.4, −5.0)	−11.2 (−14.0, −8.5)

a per 100 births.

b between post-BB rates and expected rates post-2004 (assuming the policy did not occur).

BB = baby bonus.

We assumed the outcome rates followed negative binomial distribution to account for over-dispersion in the data and used segmented regression analyses to measure the impact of the baby bonus [7]. The regression models included a term for the baby bonus policy implementation, which represented the first 12 months from the beginning of the 2^nd^ quarter of 2004 (April 2004-March 2005), during which the baby bonus was announced. This period was excluded from the time series analysis to account for the duration of pregnancy.

We estimated the pre-BB and post-BB average quarterly rates using the segmented regression models and compared the post-BB rates with the expected rates post-2004, calculated from the model as the projection of pre-BB trends under the assumption that no intervention occurred [7]. Rate differences between the post-BB and post-2004 average quarterly rates and their respective percentage changes (including 95% confidence intervals) were calculated for overall birth rates and separately by maternal demographics (age, area-based SES, and residential remoteness) and birth characteristics (parity, funding source, hospital type, mode of delivery and length of hospital stay). All analyses were performed using the statistical software SAS version 9.1 (SAS Institute Inc., Cary, NC, USA).

## Results

**Figure 1:**
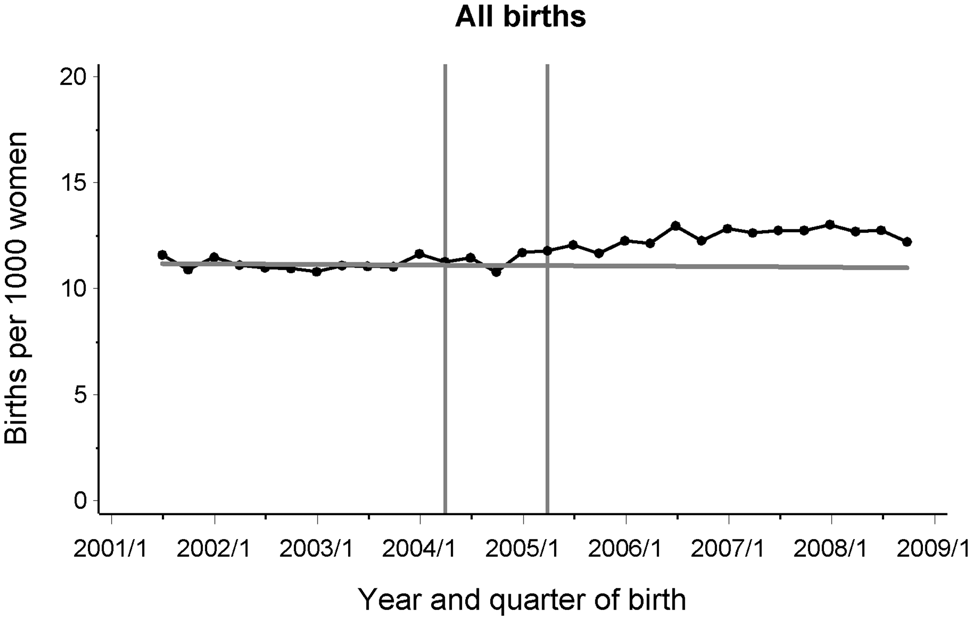
Estimated quarterly birth rates in WA during July 2001 to December 2008. Vertical lines represent the period of the baby bonus implementation (April 2004-March 2005) that was excluded from the analysis. Horizontal line represents the trend line of the pre-baby bonus period, which was projected until 2008 assuming that no policy implementation occurred.

We included 200,659 birth admissions in this study that occurred from July 2001 to December 2008 in WA. Figure 1 shows the quarterly birth rates from July 2001 to December 2008 in WA including the pre-BB trend-line projected until 2008, which represents the expected rates for the post-2004 period assuming the policy had not been introduced. The birth rates in WA rose from 11.3 births per 1000 women in the second quarter of 2004– before the baby bonus was introduced – to an ultimate high of 13.0 births per 1000 women in the first quarter of 2008 (Figure 1).

The average quarterly birth rates for the pre-BB and post-BB periods and the differences between the post-BB rates and the rates that would have been expected at the same time had the policy not occurred (post-2004) are presented in Tables 1 and 2. Following the baby bonus introduction, overall birth rates increased by 12.8% (11.3–14.5) relative to expected rates (Table 1 and Figure 1). When the change in birth rates was estimated separately by maternal demographics (Table 1), the results showed that relative to expected rates, this increase was greatest in mothers aged 20–24 years (26.3%, 95% CI = 22.0−30.6) and mothers living in areas of high residential remoteness (32.4%, 95% CI 30.2−34.6). Furthermore, when the birth rates were calculated separately by birth characteristics (Table 2), the overall birth rate increase was greatest in mothers having a third or a fourth child (1.6% and 2.2%), mothers giving birth as public patients (1.5%), mothers giving birth in public hospitals (3.5%), mothers who required vacuum/forceps extraction during delivery (16.9%) and mothers who had infants who stayed less than four days in hospital following birth (16.6%). Interestingly, birth rates to private patients decreased by 4.3%, private hospital births decreased 6.3%, caesarean sections without labour decreased by 17.9% and births where the infant stayed longer than 3 days in hospital decreased by 11.2% following the baby bonus implementation, relative to expected rates. Minimal difference was seen in the birth rate increase between mothers living in areas of high or low SES, relative to expected rates.

## Discussion

In this study we investigated the influence of the Australian baby bonus introduction on birth rates in WA. Our results indicated that the baby bonus may have served as an incentive particularly for women aged 20–24, women having their third or fourth child and women living in outer regional and remote areas. We found that the increase in birth rates following the baby bonus was greatest in mothers who gave birth in public hospitals or as public patients, for mothers who gave birth vaginally, but with assistance, and for infants who stayed less than four days in hospital following birth.

This study used population-based, routinely collected administrative hospital and birth data from WA. We were able to study almost the complete birth information in WA for the time period under study since we received de-identified data from the WA Department of Health for 99.99% of all recorded births for the entire state of WA. Despite obvious strengths relating to the population-based design, we cannot be certain that our findings are due to the baby bonus implementation alone. Some have suggested that the increase in birth rates following the baby bonus introduction could have been explained at least in part by partnering behaviour, economic conditions, unemployment rates, women’s educational attainment, labour force participation, and birth cohort effects [8], [9]. Also, information regarding the proportion of women who applied to receive the baby bonus maternity payment was not available for this study. It is likely however that the majority of mothers eligible for this payment did apply since information pertaining to this payment is widely available at birthing hospitals. Supporting this are findings reporting that despite the fact that the policy being announced only seven weeks before implementation, more babies were born on the 1^st^ July 2004 than any other date in the past 30 years in Australia with over a thousand births ‘moved’ so the parents would be eligible for the payment [10].

Three scientific articles have been published reporting on the effects of the baby bonus introduction on birth rates in Australia [3], [4], [5]. Lain et al. compared NSW birth rate trends with expected trends and found that the greatest birth rate increase was for vaginal births in public hospitals [5] and young women having their second or third child [4]. Langridge et al. compared the birth rate increase from 2004 to 2006 between different maternal characteristics groups and found that women aged 20–29 years and living in areas of higher SES contributed the most to the increase [3]. Our findings supported these previous findings, although we found only a minimal difference in birth rate increase between high and low SES areas, with a tendency towards a higher birth rate increase in high SES areas.

Our findings indicate that the baby bonus may have served as an incentive particularly for women aged 20–24, women having their third or fourth child and women living in outer regional and remote areas of Australia. Given that younger women are less likely to hold PHI than older women [11] and that private hospitals are relatively uncommon in remote Australia [12], it is perhaps not surprising that the birth rate increase was particularly evident for public patients whereas birth rates in private patients decreased. Our findings also showed that vaginal deliveries, caesareans with labour and births with shorter hospitals stays increased, whereas caesareans without labour and births with longer hospitals stays decreased. These findings are likely to reflect the much lower rate of caesarean deliveries without labour in public hospitals compared with private [11]. Furthermore, previous studies have found that length of hospital stay following birth is generally shorter in public hospitals than private hospitals in Australia [13], [14], as well as for other forms of midwifery-led care internationally [15]. It is also likely that our findings are due to the lower probability of other obstetrics interventions in the public sector since early postnatal discharge has been found to be associated with lower levels of obstetric intervention [14].

Public hospitals in Australia have been under pressure during the last couple of decades with medical workforce shortages including shortages of midwives and obstetricians [16], [17]. In an attempt to address the decline in PHI memberships among the Australian population and thus relieve pressure on public hospitals the then Australian government introduced strong tax-incentives and penalties on PHI premiums if taken out after 30 years of age in 1997–2000 to encourage the uptake of PHI [18], [19]. Our previous findings showing a decline in birth rates for public patients and an increase in birth rates for private patients after 2000–2001 [20] support previous findings reporting that these policy reforms appear to have been successful in relieving the pressure on public hospitals, particularly among more affluent Australians [21]. According to our current findings however, it appears that the baby bonus introduction may have counteracted the success of the 1997–2000 policy reforms by increasing the pressure on Australian birthing public hospitals, particularly in outer regional and remote areas.

### Conclusions

Our results indicate that following the introduction of the baby bonus maternity payment in Australia in 2004, birth rates increased particularly in mothers aged 20–24, mothers having their third or fourth child and mothers living in outer regional and remote areas. The birth rate also increased for mothers giving birth in public hospitals. Considering the reported pressure on Australian public hospitals and ongoing staff shortages, including difficulty in recruiting and retaining obstetricians in rural areas [16], the policy reform may have contributed to and increased the pressure and staff shortages in Australian public hospitals.
